# Correcting anisotropic intensity in light sheet images using dehazing and image morphology

**DOI:** 10.1063/1.5144613

**Published:** 2020-07-01

**Authors:** Tanveer Teranikar, Victoria Messerschmidt, Jessica Lim, Zach Bailey, Jung-Chih Chiao, Hung Cao, Jiandong Liu, Juhyun Lee

**Affiliations:** 1 Joint Department of Bioengineering, UT Arlington/UT Southwestern, Arlington, Texas 76010, USA; 2 Department of Electrical Engineering, Southern Methodist University, Dallas, Texas 75205, USA; 3 Department of Electrical Engineering, UC Irvine, Irvine, California 92697, USA; 4 Department of Pathology and Laboratory Medicine, University of North Carolina, Chapel Hill, North Carolina 27599, USA

## Abstract

Light-sheet fluorescence microscopy (LSFM) provides access to multi-dimensional and multi-scale *in vivo* imaging of animal models with highly coherent volumetric reconstruction of the tissue morphology, via a focused laser light sheet. The orthogonal illumination and detection LSFM pathways account for minimal photobleaching and deep tissue optical sectioning through different perspective views. Although rotation of the sample and deep tissue scanning constitutes major advantages of LSFM, images may suffer from intrinsic problems within the modality, such as light mismatch of refractive indices between the sample and mounting media and varying quantum efficiency across different depths. To overcome these challenges, we hereby introduce an illumination correction technique integrated with depth detail amelioration to achieve symmetric contrast in large field-of-view images acquired using a low power objective lens. Due to an increase in angular dispersion of emitted light flux with the depth, we combined the dehazing algorithm with morphological operations to enhance poorly separated overlapping structures with subdued intensity. The proposed method was tested on different LSFM modalities to illustrate its applicability on correcting anisotropic illumination affecting the volumetric reconstruction of the fluorescently tagged region of interest.

## INTRODUCTION

For understanding dynamic processes taking place at the cellular and molecular level, it is necessary to perform spatiotemporal volumetric analysis.^1^ Among non-invasive imaging modalities, light-sheet fluorescence microscopy (LSFM) is emerging as a powerful tool for studying developmental biology due to its highly advantageous capabilities such as short pixel dwell time while still being able to capture a high dynamic range.^2^ However, LSFM images with Gaussian illumination may suffer from limitations in the form of stripe effects and non-homogenous fluorescence intensity due to photon refraction through a heterogeneous scattering medium.^3^ Hardware solutions through dual-side and multidirectional selective plane illumination microscopy (mSPIM) have assisted to overcome these issues in conjunction with resonant mirrors.^4^ The advent of tissue clearing techniques has also helped to minimize tissue scattering by removing light scattering endogenous pigments and lipid membranes that may otherwise affect the laminar light sheet and cause tissue opacity.^5^ In addition, the oblique scanning method facilitated a major advancement in image sub-voxel resolution using a low-power objective lens.^6^ Although myriad solutions have been developed, it remains a challenge to obtain an impeccable distinction between the background and foreground due to interference from unfocused fluorescence in the axial direction. Primarily, refractive index mismatch within the tissue and mismatch between the tissue with the embedding medium cause photon scatter outside the focal volume.^7^ This leads to sample blurriness axially and laterally,^8^ hindering the accuracy of volumetric reconstruction, and is further exacerbated for a low numerical aperture (NA) objective lens that has a smaller solid angle limiting photon capture.^9^ As a result, underwater imaging requires pre-processing to maintain spatial homogeneity in the reconstructed volume.

Anisotropic intensity correction through intensity distribution approximation, based on scattering propagation models, has been implemented using conventional techniques such as histogram modeling.^10^ Such methods effectively increase the intensity dynamic range of an attenuated image, but they are susceptible to saturating bright pixel neighborhoods where evanescent scattered light flux is on the order of 10–100 greater than the incident light field.^11,12^

Biomedical applications require the critical object in the region of interest (ROI) to be aberration free and unaffected by the ambient medium. The dehazing method based on the dark channel prior (DCP) algorithm is an illumination correction model developed for correcting haze affected images captured in an outdoor environment.^13^ In this paper, we applied this method to LSFM images by assuming that the light propagation model is based on the underwater medium instead of air.^14–17^ Previous studies have indicated that the scattering coefficient of mounting media (water) and atmospheric light propagation is a function of wavelength.^18^ For the underwater condition, scattering depends on the dissolved particulate cross sectional area,^19^ whereas the composition of molecular gases determines scattering in the air.^18^ Moreover, the dehazing method possesses a high sensitivity to noise as the algorithm corrects attenuation in the aberrated image induced by medium turbidity along the line-of-sight (LOS) propagation.^13^

Translation of the mechanical stage along the depth-of-view (DOV) and lens aberration may result in phase delay or a focal point shift along the optical axis,^7^ influencing the diffraction-limited point spread function (PSF) resolution. By assuming depth invariancy,^20^ we have attempted to apply the dehazing method to recover the asymmetricity of the PSF intensity distribution. Using this method, we were able to resolve sub-pixel details along DOV (axial domain) and field-of-view (FOV) (lateral domain) without employing a high numerical aperture (NA) lens or using a speckle-based near field aperture projection of the PSF.^21^

The use of morphological operations based on isotropic structuring elements has assisted edge restoration in uneven contrast images in imaging modalities, such as x ray and CT (computed topography), which may be affected by different tissue absorption coefficients.^22,23^ By utilizing image reconstruction operations such as opening and closing, aberrations affecting the edges such as discontinuities or gaps can be corrected to maintain a uniform contour.^24^ The size of the structuring element is the factor that determines the bandpass in the frequency domain and the intensity normalization.^24^

## RESULTS

### Implementation of LSFM

**FIG. 1.**
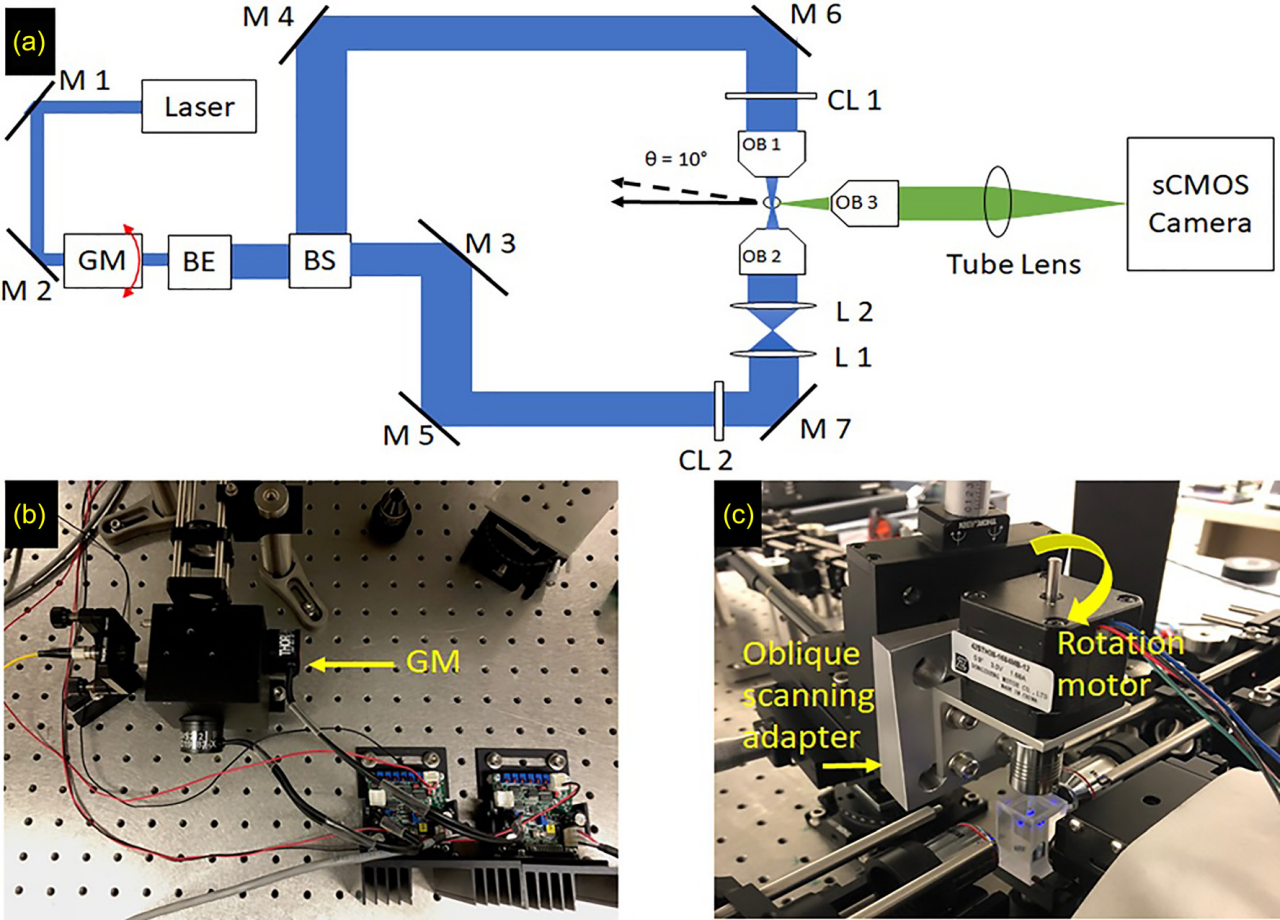
Schematic representation of the light sheet fluorescence microscopy system. (a) A 2D top view of the LSFM system, with each component showing the transformation of the beam shape. The scanning direction of the sample is indicated by the black arrow. The solid arrow represents the scanning direction for single-sided, dual-sided, and multi-view imaging. The dashed black line shows the oblique scanning angle used for super-resolution. (b) Galvanometer mirror (GM) attached to the system before the beam expander. This device is provided an input from a function generator that controls the rapid movement of light, while the sample is being scanned to reduce the stripe effect, which is a disadvantage of the regular light-sheet microscope. (c) Sample mounting stage, indicating the rotational axis used for multi-view imaging and the oblique scanning axis used in super-resolution. BE: beam expander, TS: tunable slit, BS: beam splitter, M1-7: mirror, CL1-2: cylindrical lens, L1-2: convex lens, and OB1-3: objective.

Our tunable LSFM is composed of dual-side illumination pathways integrated with multi-dimensional image fusion [Fig. 1(a)]. In the illumination path, a cylindrical lens coupled with a 4× objective lens (4X Plan Apochromat Plan N, Olympus, Tokyo, Japan) generated a collimated Gaussian light-sheet. From the calculation of the Rayleigh length (half of the confocal range), we reduced the mechanical slit to 1.99 mm to generate a 15 mm confocal range to cover the circumferential length of 4 days post-fertilization (dpf) zebrafish. To eliminate stripe effects, we added a galvanometer mirror and optimized the system at a frequency of 2000 Hz [Figs. 1(b) and S1]. When the sample was scanned through the confocal range of the light sheet along the z-direction, the sCMOS camera (ORCA flash 4.0, Hamamatsu, Japan), located at the end of the detection path coupled with a tube lens, recorded a stack of 2D plane images along different z depths. An attachable oblique scanning adapter could be easily added for applying the voxel super-resolution technique to provide high spatial resolution from a low power objective [Fig. 1(c)].

### Anisotropic illumination correction by integrating the dehazing algorithm and background subtraction

**FIG. 2.**
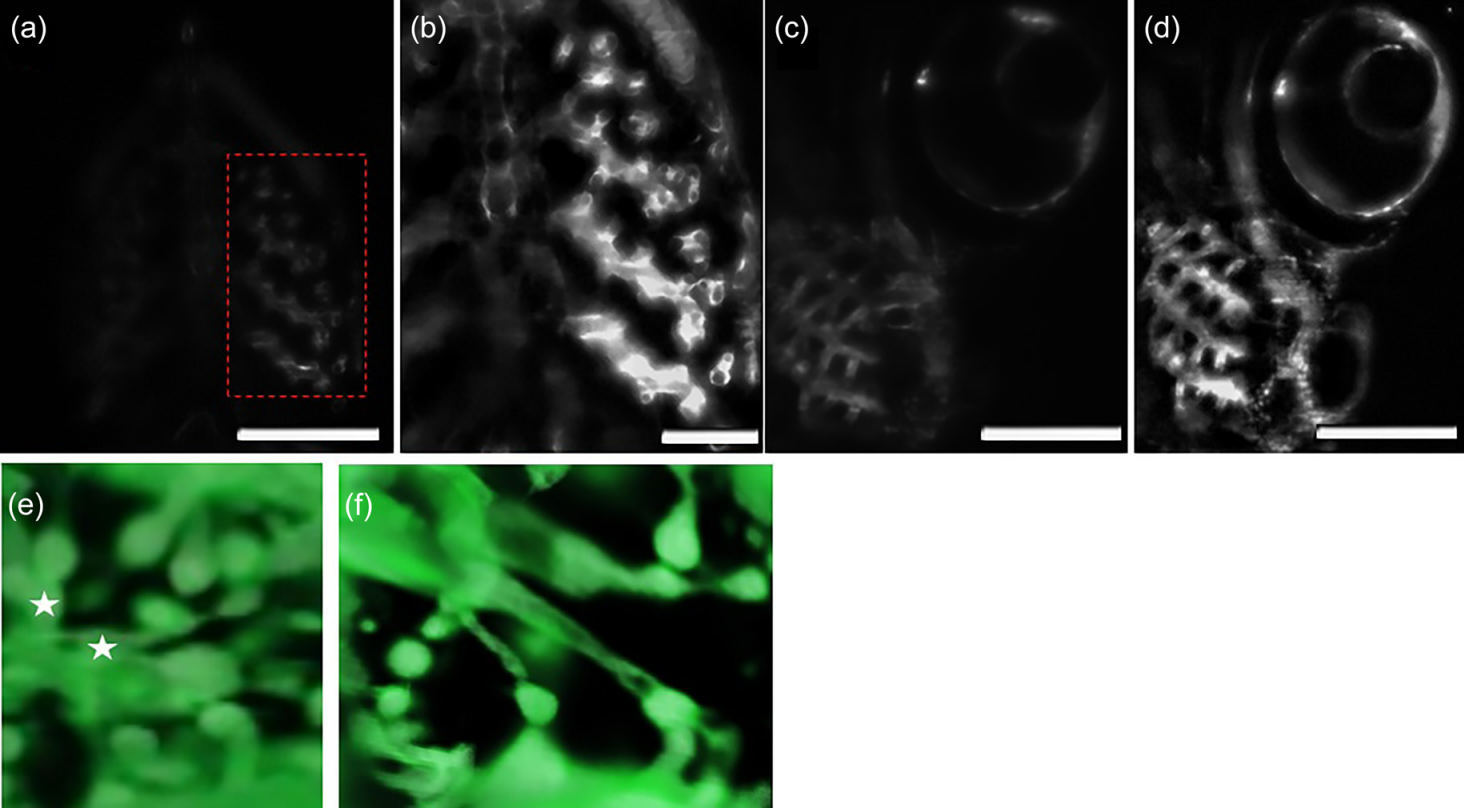
Restoring uniform intensity in the zebrafish ventral cranial vasculature by integrating dehazing and background subtraction. (a) Raw image acquired using the oblique scanning super-resolution stage. (b) Magnified view for the intensity restored ROI in the red box. After correcting attenuation through dehazing, we background subtracted the dehazed image to get rid of any diffuse light due to tissue scattering. (c) Raw image acquired using the oblique scanning super-resolution stage. (d) Intensity corrected image. (e) z-stack acquired using dual illumination before processing. White stars: The local morphology in this area suffers from poorly separated or clustered objects. This is attributed to numerous factors such as refractive index mismatch enhancing light scatter in the FOV, resulting in a saturated region of interest without clear delineation of overlapping features. (f) 3D image after processing. By restoring uniform intensity and emphasizing edges of overlapping objects, the reconstructed z stack has uniform spatial integrity for overlapping objects from different perspectives [scale bar = 200 *μ*m for (a), (c), and (d) and scale bar = 100 *μ*m for (b)].

**FIG. 3.**
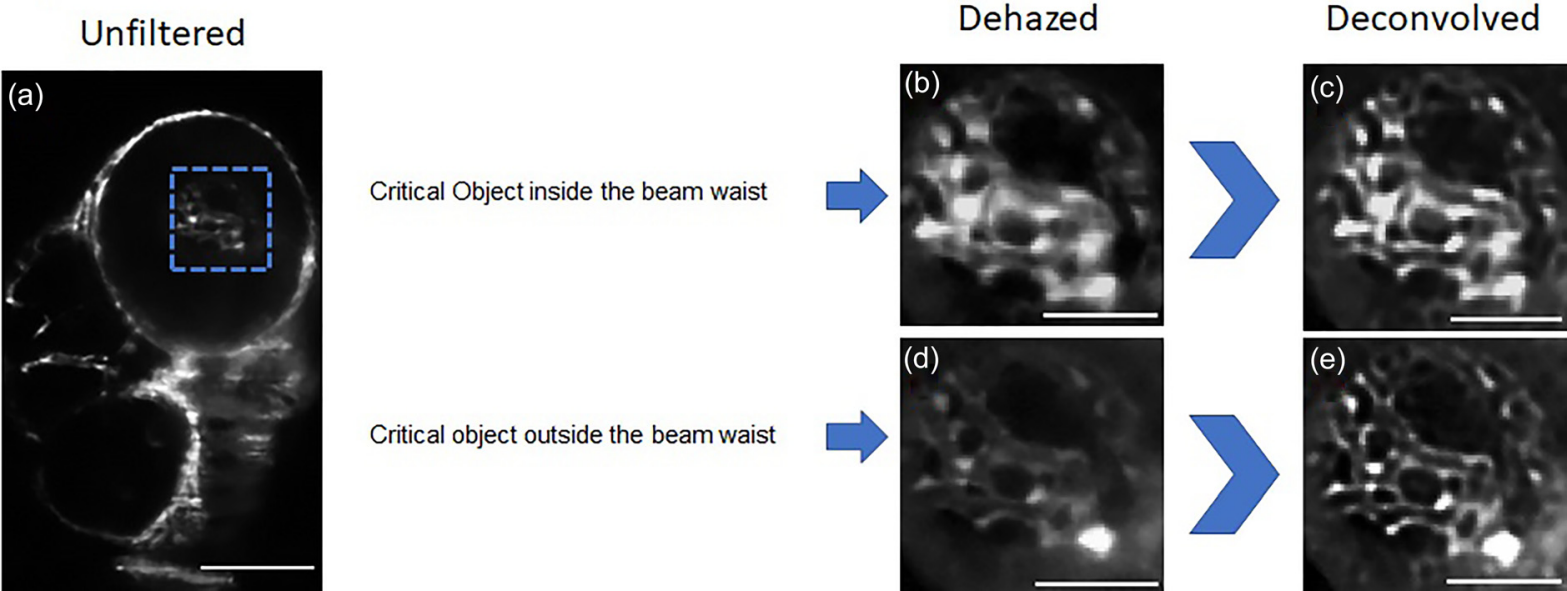
Deconvolution with dehazed PSF. (a) Unprocessed oblique scanned image. (b) Magnified perspective inside the region of interest enclosed by the blue box; dehazed image after image subtraction. (c) After deconvolving the background subtracted image (b) with the dehazed PSF, we can clearly discern overlapping depth features that are blurred due to a gradient refractive index along with the axial direction. (d) For testing the performance of the engineered PSF, we performed deconvolution on an attenuated image frame where the light sheet has passed through the critical object, so as to compare the result with a saturated image frame (b) when the critical object in the FOV is exactly perpendicular to the incoming light sheet. (e) The deconvolution process effectively restores definition to the contour inside the region of interest that may be affected by absorption or tissue scattering. To get rid of any unwanted noise that was introduced by the deconvolution process, we applied an edge preserving bilateral smoothening filter to the deconvolved images (c) and (e) [scale bar = 200 *μ*m for (b)–(e) and scale bar = 100 *μ*m for (b)–(e)].

Even after using a FEP (fluorinated ethylene propylene) tube to match the refractive indices of water and agarose, different regions of the reconstructed zebrafish anatomy suffered from attenuation of light [Figs. 2(a), 2(c), 3(a), 5(b), 5(e), 6(a), S2(a), S2(c), S3(a), S8(a), and S8(d)]. To restore uniform radiance across the attenuated image, we performed dehazing based on the dark channel prior (DCP) algorithm. The dehazing operation restores object radiance at zero viewing distance while taking into consideration atmospheric light and the transmission distance. Hence, it corrects zero intensity crossings of overlapping translucent objects without any loss of information or addition of spurious noise [Figs. 6(c), S4(a), and S4(c)].

However, dehazing based on DCP does not consider endogenous fluorescence in the focal plane due to forward scatter. Due to refractive index mismatch, light scatter takes place through the specimen in a direction perpendicular to the camera. Therefore, the dehazing method is prone to restoring unrequired background autofluorescence [Figs. 2(e), 6(c), and S4(a)]. To improve our result, we performed background subtraction of the dehazed image using a rolling ball averaging algorithm to isolate the fluorophore tagged vasculature in the image FOV and remove out of focus objects in the image plane [Figs. 2(b), 2(d), 3(b), 3(d), 6(d), S3(b), S4(b), and S4(d)].

### Correcting PSF intensity distribution using DCP

An improvement in the axial and lateral resolution was observed in the attenuated PSF after dehazing with a shift in the central peak intensity (Fig. S5). We concluded that the skew for the lateral and axial resolution graphs reflects the anisotropic shape of the bead in the xy domain as is reflected on the intensity profile plots (Fig. S6), along with depth variance along the yz domain. As deconvolution is a nonlinear process, we observed salt and pepper noise in the deconvolved images for some cases. To remove any effect of random noise introduced by deconvolution, an edge preserving bilateral smoothening filter was applied. Deconvolution of the background subtracted image using the dehazed PSF shows promising results for symmetric restoration of objects in the focal plane [Figs. 3, 6(e), and S3(c)].

### Using isotropic structuring elements to resolve edge features

The final step in the post-processing pipeline compromises top-hat and bottom-hat morphological transforms based on image opening and closing, to accurately resolve overlapping features and edge discontinuities [Figs. S4(e) and S4(f)].

**FIG. 4.**
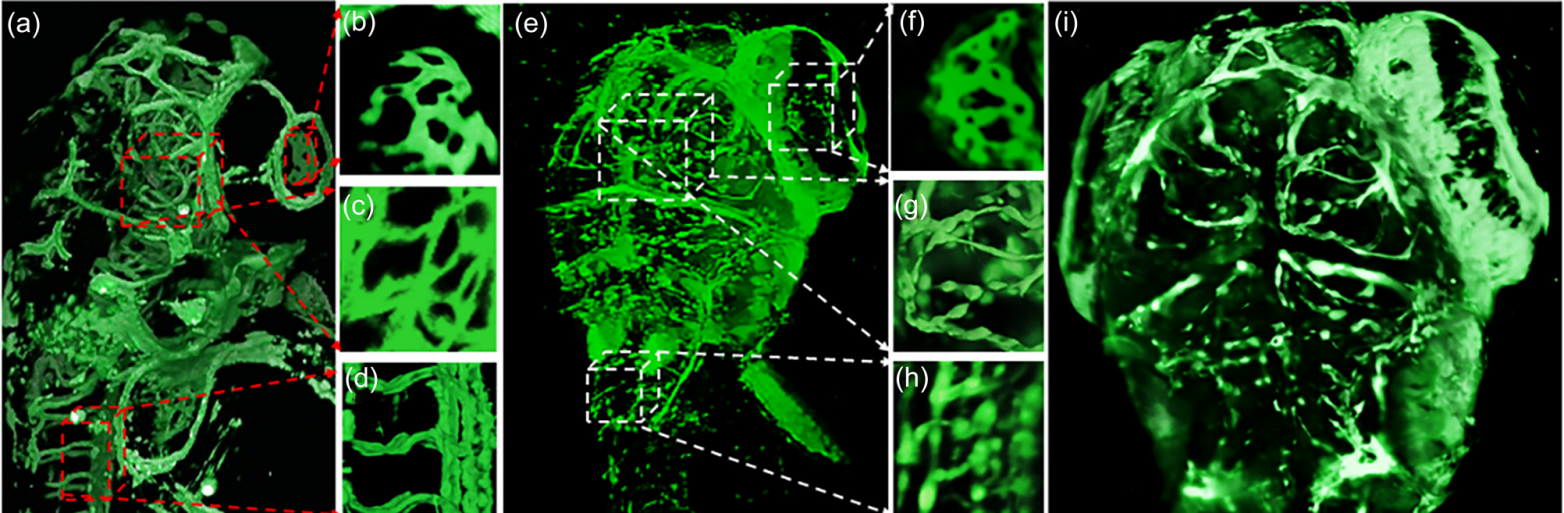
Comparison between the 3D structure of confocal modality and LSFM. (a) Z-stack acquired using a confocal microscope with the zoomed in perspectives for the eye (b), dorsal vasculature (c), and the tail (d). (e) and (i) Z-stacks acquired from the dual sided LSFM setup with the magnified regions of eye and dorsal vasculature [(f)–(h)] for comparison. We can observe better axial resolution of the light sheet volume reconstructed after processing, illuminated by sheet excitation as opposed to raster scanning point illumination for the confocal modality.

**FIG. 5.**
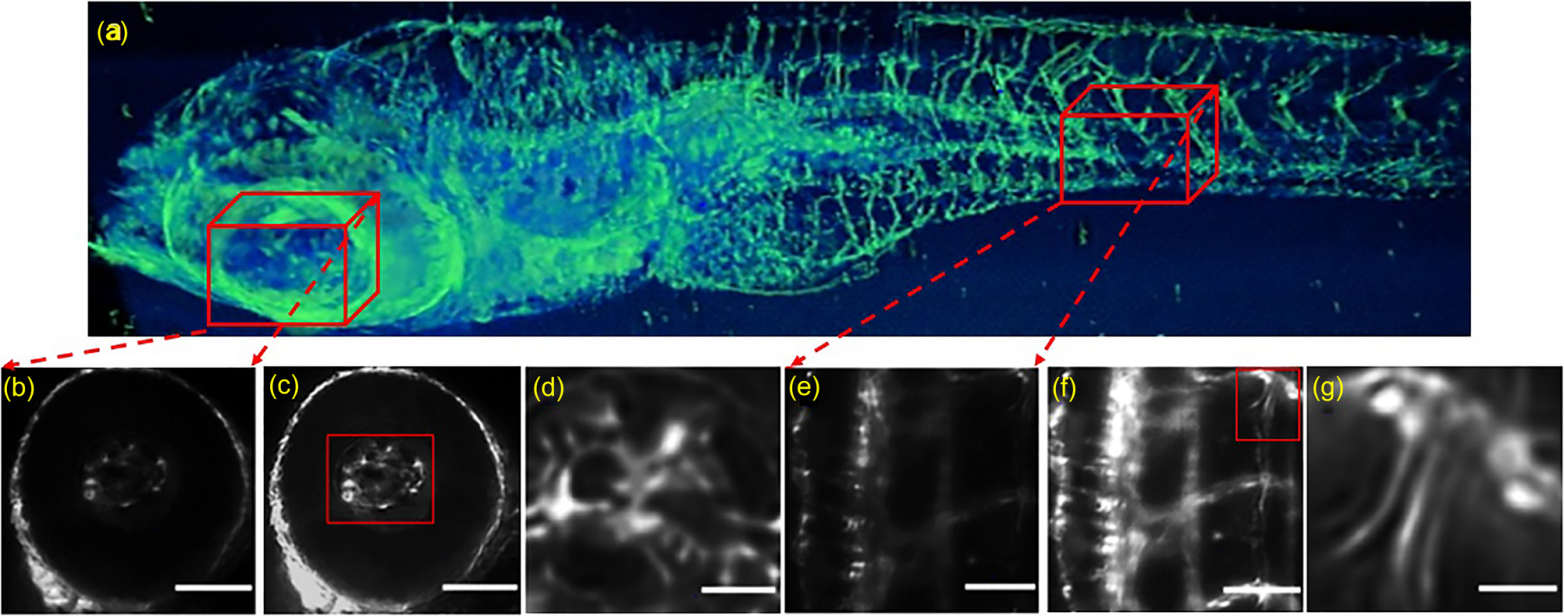
Oblique scanning based super-resolution image. (a) Orthographic 3D reconstruction of 4 dpf zebrafish vasculature. [(b) and (e)] High resolution images were acquired from a large field-of-view. [(c) and (f)] Intensity corrected images with highly resolved boundaries between structures were obtained after applying the proposed algorithm to (b) and (e), in that order. [(d) and (g)] Zoomed-in images obtained from (c) and (g) (red squares) show the details of the super-resolution method [scale bar = 100 *μ*m for (b), (c), (e), and (f) and scale bar = 30 *μ*m for (d) and (g)].

**FIG. 6.**
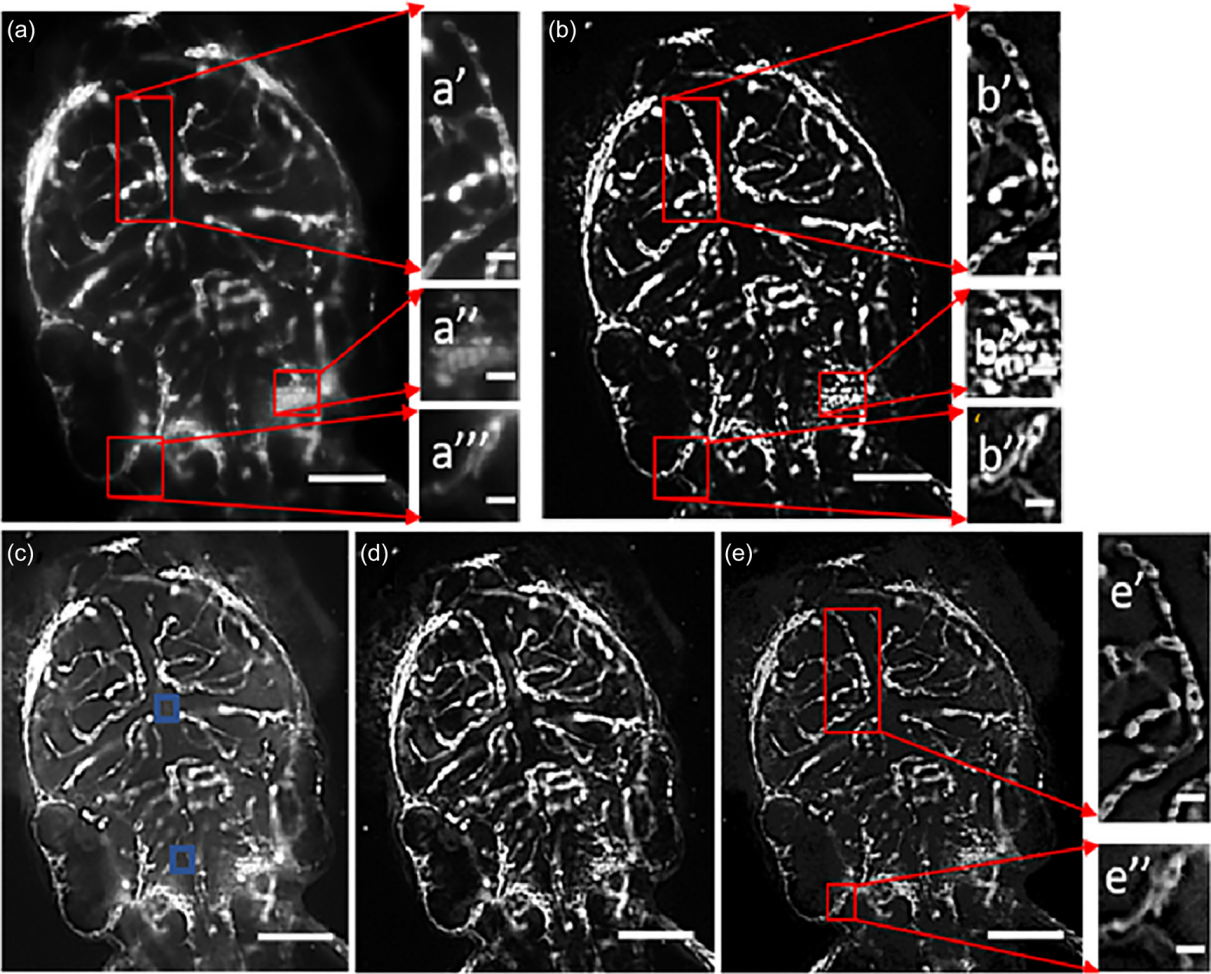
Image processing algorithm. (a) Original image with its zoomed in regions of middle mesencephalic central artery (a′), primordial hindbrain channel (a″), and posterior cerebral vein (a‴), respectively. (b) Super-resolved image with its magnified perspective views of highlighted blood vessels (b′, b″, and b‴). (c) After the processed dehazing technique, autofluorescence or tissue scattering has been amplified together with the fluorescent signal. (d) After dehazing, background subtraction was applied to increase the fluorescent SNR of zebrafish vasculature. (e) Using dehazed PSF deconvolved to resolve the image of fine vascular system [(e′) and (e″)] [scale bar = 200 *μ*m for (a)–(e) and scale bar = 50 *μ*m and (a′)–(a‴), (b′)–(b‴), (e′), and (e″)]. Blue square: local pixel patch showing autofluorescence.

After obtaining the deconvolved image, we applied the top-hat transform to the dehazed image. The top-hat transform is based on image erosion, which is utilized to remove weakly connected regions of interest (ROI) and also image dilation to emphasize and fill-in image boundaries, in that order. Top-hat transform assists by removing any pixel discontinuities induced by background subtraction and effectively isolates foreground objects in the image plane. The bottom-hat transform is based on image closing to reinforce depth details, which are blurred out and out-of-focus due to autofluorescence affecting the axial resolution. The final image was reconstructed by adding the image subtraction of the top-hat and bottom-hat transform results to the dehazed image. Isotropic structural integrity was achieved across different image dimensions [Figs. 2(f), 4(f)–4(h), 4(i), 5, 6(b), 6(b′)–6(b‴), S2(b), S2(d), S3(d), and S8].

### Application of the proposed method to various LSFM techniques

We acquired images using different LSFM modalities such as single/dual illumination SPIM, multiview SPIM with dual illumination, and the oblique scanning super-resolution stage integrated with dual illumination. To construct the dual illumination arms orthogonal to the detection camera, a 50:50 beam splitter was used to separate the incoming Gaussian laser spot into two equally intense laser beams [Fig. 1(a)]. The dual-sided LSFM increases the illumination efficiency as a result of which depth features can be better distinguished [Fig. 6(a)], as opposed to single sided illumination [Fig. S3(a)].

For resolving the vasculature of the zebrafish at different scales without loss of spatial homogeneity, we acquired images using an oblique scanning stage to apply the voxel super-resolution algorithm. By applying our enhancement algorithm with the image processing pipeline, minute structures, such as optic arteries and veins on the head, and intersegmental vessels on the tail were reconstructed clearly with a 4× objective lens [Fig. 6(b)]. Taking advantage of our processing, we reconstructed a multi-view fused stack from the processed images that were captured by an oblique scanning stage. Multi-view fusion is utilized to overcome opacity or translucency, which affects spatial homogeneity due to light scattering, by adding images from different angles (Fig. S9). The fused image exhibits superior depth reconstruction of different orthogonal perspectives in conjunction with the oblique scanning technique (Fig. S10).

### Evaluation parameter of image restoration

For restoration quality assessment, the structural similarity index (SSIM) full reference method was used by using a histogram adjusted raw image as reference [Fig. S11(d)]. The lower/upper 10% of intensity distribution was saturated in order to enhance under exposed structures. Using the reference image, we compared the loss/degradation by our proposed method using dehazing followed by background subtraction [Fig. S11(b)] and a contrast limited adaptive histogram equalization (CLAHE) method [Fig. S11(c)]. We plotted the local SSIM maps, based on the per-pixel value, and calculated the global SSIM value as well [Figs. S11(e) and S11(f)]. Both the proposed method and the CLAHE method scored 0.86 on the global SSIM value (lower limit= −0.54/upper limit= +0.93, scores reported using original model development). Adjusting parameters for either algorithms yielded similar global SSIM values with the lowest score being +0.1 and the best score being 0.9. We also calculated the peak signal to noise ratio (PSNR) to understand improvement in terms of noise reduction during each step of the processing. For PSNR evaluation, an oblique scanned raw image was used for comparison (Fig. S12).

## DISCUSSION

In this paper, we demonstrated the applicability of the DCP algorithm for restoring isotropic intensity in the FOV and morphological operations for correcting diffraction-limited volumes with non-homogenous spatial homogeneity. We observed some intrinsic limitations in the single/dual side LSFM images acquired at the detection focal plane that affected isotropic reconstruction of the z stack. The images composed of local pixel patches were innately high in photon count with reduced visibility [Fig. 6(a)]. For this study, we assumed that scattered wavelengths in these regions are longer than the excitation wavelengths as seen in Raman Stokes scattering and are non-propagating.^11^ This light scatter spreads laterally across the tissue but does not participate in the far field image formation. It decays evanescently in a direction perpendicular to the surface with the intensity distribution being amplified by an order of 10–100 in the evanescent field region.^11^ Coupled with a laser intensity beyond a certain threshold,^7^ it can cause optical distortions for any particulates that are less than half the input wavelength. As a result, out of focus tissue planes in the evanescent light field are illuminated. Due to this limitation, dull/low light patches with relatively uniform colocalized zero intensity crossings appear saturated due to over estimation of haze thickness (Figs. S13 and S14).

The capability of an interrogating light sheet to illuminate the sample and the ability of the emitted light to escape the sample and reach the detection camera constitute important factors for determining image resolution. Using a low NA focusing objective can compromise the excitation efficiency of the focused beam waist^7,18^ (Fig. S15). As LSFM involves light propagation through media with differing refractive indices, it is also necessary to develop or select intensity correction algorithms that account for these changes in optical properties of the media. Even when refractive index mismatch between the FEP tube and mounting media is neglected, light scattering occurs due to the variation in tissue refractive indices.^25–29^ Tissue birefringence due to the tissue refractive index is a function of the density, resulting in a gradient refractive index.^30^

Tissue transparency in animals is a function of surface irregularities at the cellular/molecular level, which affect the potential of tissue to reflect light. Using the Weber formula for defining contrast (Cd) of a transparent animal, we get^28^

Cd=((Ld − Lb)/Lb), (1)

where *Ld* is the radiance of the object viewed at a distance in the underwater medium and *Lb* is the background radiance,^28^

$$\mathrm{Ld}=(\mathrm{Lo}e^{\left(-cd\right)}+\mathrm{Lb}(1-e^{\left(-cd\right)})),$$ (2)

where *Lo* is the inherent radiance at zero viewing distance, *c* is the beam attenuation coefficient (wavelength is a function of the depth), and *d* is the distance of the object to the viewer. Here, the detected object radiance (*Ld*) depends on object irradiance at the point of focus and the effect of atmospheric light scatter along the LOS. As we are using along working distance air lens with low NA, we can infer from the above equation that as the viewing distance is increased, inherent object radiance will decrease and the scattered light (*Lb*) will dominate.

For a transparent animal, the inherent radiance consists of two parts, namely, the background radiance that is transmitted through the animal and the light scattered toward the viewer; hence,^28^

$$\mathrm{Ld}=(\mathrm{LbT}e^{\left(-cd\right)}+\mathrm{LdS}e^{\left(-cd\right)}+\mathrm{Lb}(1-e^{\left(-cd\right)})),$$ (3)

where *T* represents transmission through the animal and *S* is a fraction of the environmental light scattered to the observer.

Comparing equation (3) with the Dehazing equation,^13,14^ we get

$$I\left(x\right)=J\left(x\right).t\left(x\right)+A\left(1-t\left(x\right)\right),$$

where *I*(*x*) is the attenuated image recovered using the camera (Fig. S15), *J*(*x*) is the original scene irradiance that has a multiplicative effect with the transmission distance, *t*(*x*) (Fig. S15), and *A* is the ambient/background light. Therefore, we infer that the DCP method does not take into account the background radiance, *Lb*, which is transmitted through the illuminated portion, *T*, of the object. To counter this problem, we performed the background subtraction operation on the dehazed image to remove innately present autofluorescence in the surrounding tissue.

Fluorescent images captured at higher frame rates suffer from poor distinction between the fluorescently tagged marker and background noise. To further elucidate depth details and separate overlapping edges, we used top-hat and bottom-hat morphological transforms based on flat structuring elements. As morphological operations are non-linear operations based on spatial ordering of the pixel coordinates and not the pixel intensity values, these methods can be applied efficaciously to grayscale turbid media images. To maintain isotropic kernel convolution values in all directions, we used a sphere-shaped structuring element that was greater than the smallest region-of-interest (ROI) for all the morphological operations.

As the PSF of diffraction-limited microscopy is depth and space invariant with respect to the detection optic axis, we applied the dehazing algorithm as a PSF intensity correction model. An intensity correction of the recorded emission intensity for a fixed dipole, or fluorescent bead, was implemented by assuming that the intensity will vary according to the random orientation of the bead (Fig. S9). Assuming anisotropic photon collection efficiency, the intensity will experience a higher degree of exponential decay for long working distance objective lenses.

In summary, with the help of our image processing, we were able to overcome intrinsic LSFM limitations and improve the spatial resolution of the turbid media image. The dehazing model was used to enhance subdued intensity peaks and thus restore attenuation caused due to propagation through an opaque medium or light sheet skew. Using morphological operators, we were able to localize peaks and valleys in the local image structure in order to resolve diffraction limited structures affected by light scatter or stripe artifacts. At the same time, we were able to capitalize on the advantages provided by the LSFM modality, namely, the wide FOV provided by the low NA infinity corrected objective lens, and acquire zebrafish z-stacks with short acquisition time. This research will benefit *in vivo* developmental biology studies, visualized using fluorescence modalities that require accurate volumetric reconstruction for further analysis.

## METHODS

### Preparation of zebrafish imaging

The experiments were performed in compliance with the approval from the UT Arlington Institutional Animal Care and Use Committee (IACUC) protocol (#A17.014). The transgenic lines, *Tg*(*flk:gfp*), were raised at the UT Arlington Aquatic Animal Core Facility. The flk promoter-driven green fluorescent protein (GFP) from the *Tg*(*flk:gfp*) zebrafish line was expressed in vascular endothelial and endocardial cells.^31^ To maintain the transparency of zebrafish embryos, the medium was supplemented with 0.0025% phenylthiourea (PTU) to suppress pigmentation at 20 h post fertilization (hpf). At 4 dpf, live transgenic zebrafish embryos were anesthetized in 0.05% tricaine and immersed in 37 °C, 0.5%, low-melt agarose for imaging. Prior to agarose solidification, the embryos with low-melt agarose were transferred to a fluorinated ethylene propylene (FEP) tube to match the refractive index (1.34) to that of water (1.33).

### LSFM setup

Our tunable LSFM was set up to be able to use single side illumination, dual-side illumination, multi-view fusion, and oblique scanning for the VSR (Voxel super resolution) technique. To cover the entire width of the zebrafish, we calculated the confocal range from double of the Rayleigh length ($z_{R}$), which is

$$z_{R}=\frac{\pi w_{0}^{2}}{\lambda },$$

where λ is the wavelength of our laser (473 nm) to illuminate the green fluorescent protein in the transgenic zebrafish line and $w_{0}$ is the beam waist, which is the thinnest focal point of the Gaussian beam.

The width of the light-sheet w(z) along the z-axis can be calculated as follows:

$$w(z)=w_{0}\sqrt{1+{\left(\frac{\lambda \cdot z}{\pi \cdot w_{0}^{2}}\right)}^{2}.}$$

We also adjusted the slit width to control the light-sheet thickness at the sides of the sample of $\sqrt{2}w(z)$, which is the thickness of the light-sheet under the Rayleigh length.

### Voxel super-resolution

The technique of super-resolution by using oblique scanning follows the principle of the function of pixel super-resolution techniques in a 2D spatial domain. Super-resolved estimate *I* is most consistent with multiple measurements *P_k_* after a series of degradation operators.^32^ Here, we solved *I* via minimizing the following cost function:

$$\hat{\underline{I}}=\underset{\underline{I}}{\text{ArgMin}}\left[\sum_{k=1}^{n}\rho (P_{k},D_{k}O_{k}S_{k}\underline{I})\right],$$

where ρ is the difference between the model and measurements. *S_k_* is the geometric motion operator between HR (high-resolution) estimate *I* and *k^th^* LR (low-resolution) *P_k_*, the point-spread-function of the oblique scanning LSFM system is modeled by the blur operator *O*, and *D_k_* represents the decimation operator that models digital sampling of the camera. Theoretically, the computation estimates a unique HR image, which has maximum likelihood to the LR inputs after given degradations *S_k_*, *O_k_*, and *D_k_* are applied. Practically, a steepest descent method is provided to iteratively approach a converged super-resolved solution at high efficiency,

$${\hat{\underline{I}}}_{m}={\hat{\underline{I}}}_{m-1}-\beta \left[\sum_{k=1}^{n}S_{k}^{T}O_{k}^{T}D_{k}^{T}s\mathrm{ign}(D_{k}O_{k}S_{k}{\hat{\underline{I}}}_{m-1}-{\underline{P}}_{k})\right],$$

where $S_{k}^{T},O_{k}^{T},D_{k}^{T}$ represent the inverse operation of $S_{k},O_{k},D_{k}$, respectively. This maximum likelihood estimation (MLE) solving process is further described in the form of a block diagram. A detailed description can be found in our previous research.

### Image processing pipeline

The proposed method was implemented and tested in MATLAB R2019B and ImageJ. Observing better lateral resolution as compared to axial resolution, the processing was applied to every individual image slice in the z-stack (Fig. S16). The dehazing, deconvolution, and morphological operations were performed using the imreducehaze (dehazing operation), deconvlucy (point spread function deconvolution), and imtophat and imbothat (morphological operations) functions, respectively. We performed the background subtraction method in Fiji and used Amira to reconstruct the z-stack by specifying the voxel size parameters.

The dehazing method tends to amplify the autofluorescence of tissue outside the focal plane and as a result hampers the intensity restoration [Fig. 6(c)]. To avoid this artifact, we performed background subtraction on the dehazed image [Fig. 6(d)]. Furthermore, we deconvolved the background subtracted image using a dehazed point spread function [Figs. 6(e), 6(e′), and 6(e″)]. The deconvolution result is a function of the number of iterations and hence can introduce random noise. We smoothened the image after deconvolution using a Gaussian smoothening filter in MATLAB using the imgaussfilt function or alternatively an edge preserving bilateral smoothening filter in ImageJ (*Plugins > Process > Bilateral Filter*). Both methods produced similar results without any significant change. It is important to adjust the parameters of PSF dehazing and select the PSF along LOS propagation unaffected by spherical aberrations such that it does not lead to PSF oversampling causing unwanted saturation. The final image [Figs. 6(b), 6(b′), and 6(b‴)] was obtained through an image arithmetic operation as follows (Fig. S16):

(background subtracted dehazed image) – [tophat(background subtracted dehazed image) + bottomhat(dehazed image deconvolved with the dehazed point spread function)].

After reconstructing the z-stack by specifying the required voxel arguments, we were able to visualize the 3D local morphology across different scales by adjusting the transparency of the volrenGreen color map and the histogram to saturate critical objects in the 3D FOV.

(1) Dehazing: The raw images were contrast-corrected using the dehazing method based on the dark channel prior (DCP) algorithm. Image formation achieved by the dehazing algorithm is based on the following model:^32^
  $$I\left(x\right)=(J\left(x\right).t\left(x\right)+A\left(1-t\left(x\right)\right)),$$
  where *I*(*x*) is the attenuated image, *J*(*x*) is the dehazed image at the original scene of irradiance captured using the camera over LOS propagation, A is the ambient light, and t(x) is the light transmission that is unaffected by interference by the ambient surroundings.
(2) Background subtraction: The sliding paraboloid background subtraction filter in Imagej based on the rolling ball averaging algorithm^33^ was used to remove the effect of tissue autofluorescence and lateral light scatter. It is based on image subtraction of an averaged background value for each pixel from the original image by sliding a paraboloid estimated by four parabolas in four directions: x,y and two 45° directions. The recovered image contains a gradient refractive index of overlapping tissue represented as an unsigned integer in the 2D image vector. These overlapping spatial variations represented as intensity variations in local pixel neighborhoods can be effectively corrected using background subtraction as it is based on the assumption that each pixel intensity on the XY spatial image plane can be imagined as a third dimension with respect to the intensity values of other pixels in the local neighborhood. This is analogous to using non-flat structural elements for the mentioned morphological operations.
(3) Gaussian blurring filter: The deconvolution process may result in over-emphasized intensity peaks or unwanted noise amplification. To reduce this effect, we applied the following Gaussian smoothing function:
  $$G\left(x,y\right)=\frac{1}{2\pi \sigma^{2}}e^{-\frac{x^{2+y^{2}}}{2\sigma 2}},$$
  where x denotes the distance from the horizontal axis, y is the distance from the vertical axis, and σ (sigma) is the standard deviation for a 2D isotropic Gaussian convolution kernel. We specifically used the Gaussian smoothing filter, as other filters that replace the pixel values with the weighted average of neighboring pixel values may pass intensity values that may be beyond the passband (ringing oscillations). This is also because convolution with a smoothing kernel in the spatial image domain is analogous to multiplication in the frequency domain with a low pass frequency filter. Since the value of sigma (σ) determines the degree of smoothing, we used a smaller σ value to maintain a tighter roll-off for the Gaussian function and to avoid excess blurring.
(4) Morphological transformation: For grayscale image f and structuring element B,^23,24^3pt
  $$\text{Image dilation}:\delta_{B\;}f_{\left(x,y\right)}=\text{max}\{f\left(x-s,\;y-t\right)+B\left(s,t\right)|(x-s),(y-t)\in D_{f},(s,t)\in D_{B},$$
  $$\text{Image erosion}:\epsilon_{B\;}f_{\left(x,y\right)}=\text{min}\{f\left(x+s,\;y+t\right)-B(s,t)|\left(x+s\right),(y+t)\in D_{f},(s,t)\in D_{B},$$
  where (x,y) and (s,t) are the coordinate sets for grayscale image f and structuring element B, respectively, and $D_{f}$ and $D_{B}$ are the respective domains.The image opening and closing operations are defined as follows:^13^
  $$\text{Image opening}:\gamma_{\left(B\right)}(f)=\delta_{B\;}(\epsilon_{B\;}\left(f\right)),$$
  $$\text{Image closing}:\emptyset_{\left(B\right)}\left(f\right)=\epsilon_{B\;}\left(\delta_{B\;}\left(f\right)\right).$$
  The top hat transform is based on image subtraction of the original image by the opened image. This morphological operation was utilized to restore contrast in uneven illumination along the line of sight (LOS) propagation in this case. Image opening can be defined as image erosion followed by image dilation. The bottom hat transform was used to emphasize poorly illuminated depth details, and the bottom-hat image was obtained by dilating the image and eroding it. The top hat transform was employed to maintain the size of larger objects and remove smaller discontinuities, whereas the bottom hat transform was used to fill-in smaller regions. For images that were relatively unaffected by any intensity aberrations, we used the opening operation as an alternative to the top hat operation and closing operation instead of bottom hat filtering.

## SUPPLEMENTARY MATERIAL

See the supplementary material for understanding the image restoration methods in the implemented pipeline.

## Data Availability

The data that support the findings of this study are available from the corresponding author upon request.

## References

[1] Y. Ding , J. Lee , J. Ma , K. Sung , T. Yokota , N. Singh , M. Dooraghi , P. Abiri , Y. Wang , R. P. Kulkarni , A. Nakano , T. P. Nguyen , P. Fei , and T. K. Hsiai , Sci. Rep. 7, 42209 (2017). 10.1038/srep42209 28165052 PMC5292685

[2] J. Andilla , O. E. Olarte , E. J. Gualda , and P. Loza-Alvarez , Adv. Opt. Photonics 10(1), 111 (2018). 10.1364/AOP.10.000111

[3] S. Johnsen , Annu. Rev. Mar. Sci. 6, 369 (2014). 10.1146/annurev-marine-010213-135018 23987915

[4] J. Huisken and D. Y. R. Stainier , Opt. Lett. 32, 2608–2610 (2007). 10.1364/OL.32.002608 17767321

[5] D. Jing , S. Zhang , W. Luo , X. Gao , Y. Men , C. Ma , X. Liu , Y. Yi , A. Bugde , B. O. Zhou , Z. Zhao , Q. Yuan , J. Q. Feng , L. Gao , W.-P. Ge , and H. Zhao , Cell Res. 28(8), 803 (2018). 10.1038/s41422-018-0049-z 29844583 PMC6082844

[6] P. Fei , J. Nie , J. Lee , Y. Ding , S. Li , H. Zhang , M. Hagiwara , T. Yu , T. Segura , and C.-M. Ho , Adv. Photonics 1(1), 016002 (2019). 10.1117/1.AP.1.1.016002

[7] V. Crosignani , A. Dvornikov , J. S. Aguilar , C. Stringari , R. Edwards , W. W. Mantulin , and E. Gratton , J. Biomed. Opt. 17(11), 116023 (2012). 10.1117/1.JBO.17.11.116023 23214184 PMC3494495

[8] L. E. Bagge , S. T. Kinsey , J. Gladman , and S. Johnsen , J. Exp. Biol. 220 (Pt. 22), 4225 (2017). 10.1242/jeb.162362 29141882

[9] H. Gross , *Handbook of Optical Systems, Volume 1: Fundamentals of Technical Optics* ( Wiley-VCH, 2005), p. 848, ISBN: 3-527-40377-9.

[10] S. S. Sankpal and S. Sunil Deshpande , J. Eng. 2016, 2.

[11] R. R. Jones , D. C. Hooper , L. Zhang , D. Wolverson , and V. K. Valev , Nanoscale Res. Lett. 14(1), 231 (2019). 10.1186/s11671-019-3039-2 31300945 PMC6626094

[12] S. S. S. Singh Dhillon , Int. J. Innovations Adv. Comput. Sci. 5(1), 12 (2016).

[13] K. He , J. Sun , and X. Tang , IEEE Trans. Pattern Anal. Mach. Intell. 33, 2341 (2011). 10.1109/TPAMI.2010.168 20820075

[14] J. Čejka , M. Žuži , F. Bruno , D. Skarlatos , and F. Liarokapis , Front. Rob. AI 5, 1 (2018). 10.3389/frobt.2018.00092 PMC7805651 33500971

[15] M. A. Malathi V , Int. J. Eng. Adv. Technol. (IJEAT) 9(2), 280 (2019).

[16] Y. Li , H. Lu , L. Zhang , and S. Serikawa , J. Opt. Soc. Am. A 32, 886 (2015). 10.1364/JOSAA.32.000886 26366913

[17] T. M. Nimisha , K. Seemakurthy , A. N. Rajagopalan , N. Vedachalam , and, and R. Raju , in *Proceedings of the Tenth Indian Conference on Computer Vision, Graphics and Image Processing* ( Association for Computing Machinery, Guwahati, Assam, 2016), Article No. 26.

[18] M. Bass , E. W. Van Stryland , D. R. Williams , and W. L. Wolfe , *Handbook of Optics, Volume I: Fundamentals, Techniques, and Design*, 2nd ed. ( McGraw-Hill, Inc, New York, NY, 1995).

[19] M. J. Costello , S. Johnsen , K. O. Gilliland , C. D. Freel , and W. C. Fowler , Invest. Ophthalmol. Visual Sci. 48(1), 303 (2007). 10.1167/iovs.06-0480 17197547

[20] B. Kim and T. Naemura , Sci. Rep. 5(1), 9894 (2015). 10.1038/srep09894 25950821 PMC5155489

[21] X. Xie , H. Zhuang , H. He , X. Xu , H. Liang , Y. Liu , and J. Zhou , Sci. Rep. 8(1), 4585 (2018); 10.1038/s41598-018-22966-7 29545584 PMC5854624

[22] Y. Kimori , N. Baba , and N. Morone , Bioinformatics 11(373), 373 (2010). 10.1186/1471-2105-11-373 20615231 PMC2914730

[23] Y. Kimori , J. Synchrotron Radiat. 20(Pt. 6), 848 (2013). 10.1107/S0909049513020761 24121326 PMC3795542

[24] G. Wang , Y. Wang , H. Li , X. Chen , H. Lu , Y. Ma , C. Peng , Y. Wang , and L. Tang , PLoS One 9(11), e110991 (2014). 10.1371/journal.pone.0110991 25426639 PMC4245115

[25] S. Johnsen , Sci. Am. 282(2), 62 (2000). 10.1038/scientificamerican0200-80 10710790

[26] S. Johnsen and E. Widder , J. Theor. Biol. 199(2), 181 (1999). 10.1006/jtbi.1999.0948 10395813

[27] S. Johnsen , E. A. Widder , and C. D. Mobley , Biol. Bull. 207, 1 (2004). 10.2307/1543624 15315939

[28] S. Johnsen , N. J. Marshall , and E. A. Widder , Philos. Trans. R. Soc., B 366(1565), 655 (2011). 10.1098/rstb.2010.0193 PMC3049004 21282169

[29] S. Johnsen and H. M. Sosik , Limnol. Oceanogr. 48(3), 1277 (2003). 10.4319/lo.2003.48.3.1277

[30] S. Johnsen , Integr. Comp. Biol. 43, 580 (2003). 10.1093/icb/43.4.580 21680466

[31] M. E. Moghadam , J. Lee , E. Kung , H. Cao , T. Beebe , Y. Miller , B. L. Roman , C. L. Lien , N. C. Chi , A. L. Marsden , and T. K. Hsiai , PLoS One 8, e72924 (2013). 10.1371/journal.pone.0072924 24009714 PMC3751826

[32] D. Robinson and P. Milanfar , IEEE Trans. Image Process. 13, 1185 (2004). 10.1109/TIP.2004.832923 15449581

[33] S. R. Sternberg , Computer 16(1), 22 (1983). 10.1109/MC.1983.1654163

